# Characteristics of drug resistance mutations in ART-experienced HIV-1 patients with low-level viremia in Zhengzhou City, China

**DOI:** 10.1038/s41598-024-60965-z

**Published:** 2024-05-09

**Authors:** Jinjin Liu, Chaofeng Li, Yan Sun, Chaohong Fu, Shuguang Wei, Xiaohua Zhang, Jie Ma, Qingxia Zhao, Yuqi Huo

**Affiliations:** Affiliated Infectious Diseases Hospital of Zhengzhou University (Henan Infectious Diseases Hospital, The Sixth People’s Hospital of Zhengzhou), No.29, Jingguang South Road, Erqi District, Zhengzhou, 450000 China

**Keywords:** HIV-1, Low-level viraemia, Drug resistance mutations, Antiretroviral therapy, ART-experienced, Subtypes, HIV infections, Infection

## Abstract

Although most people living with HIV (PLWH) receiving antiretroviral therapy (ART) achieve continuous viral suppression, some show detectable HIV RNA as low-level viremia (LLV) (50–999 copies/mL). Drug resistance mutations (DRMs) in PLWH with LLV is of particular concern as which may lead to treatment failure. In this study, we investigated the prevalence of LLV and LLV-associated DRMs in PLWH in Zhengzhou City, China. Of 3616 ART-experienced PLWH in a long-term follow-up cohort from Jan 2022 to Aug 2023, 120 were identified as having LLV. Of these PLWH with LLV, we obtained partial pol and integrase sequences from 104 (70 from HIV-1 RNA and 34 from proviral DNA) individuals. DRMs were identified in 44 individuals. Subtyping analysis indicated that the top three subtypes were B (48.08%, 50/104), CRF07_BC (31.73%, 33/104), and CRF01_AE (15.38%, 16/104). The proportions of nucleoside reverse transcriptase inhibitors (NRTIs), non-nucleoside reverse transcriptase inhibitors (NNRTIs), protease inhibitors (PIs), and integrase strand transfer inhibitors (INSTIs) associated DRMs were 23.83% (24/104), 35.58% (37/104), 5.77% (6/104), and 3.85% (4/104), respectively, which contributed to an overall prevalence of 42.31% (44/104). When analyzed by individual DRMs, the most common mutation(s) were V184 (18.27%, 19/104), followed by V179 (11.54%, 12/104), K103 (9.62%, 10/104), Y181 (9.62%, 10/104), M41 (7.69%, 8/104), and K65R (7.69%, 8/104). The prevalence of DRMs in ART-experienced PLWH with LLV is high in Zhengzhou City and continuous surveillance can facilitate early intervention and provision of effective treatment.

## Introduction

Plasma human immunodeficiency virus-1 (HIV-1) RNA level is an important marker for clinical diagnosis of HIV infection and efficacy evaluation of antiretroviral therapy (ART). Plasma viral load (VL) in people living with HIV (PLWH) is highly correlated with the risk of HIV transmission^1^. Although standardized ART can suppress viral replication in most PLWH to a level below the lower limit of detection (< 50 copies/mL), however, in clinical settings, approximately 20–30% of PLWH present with a VL between 50 and 999 copies/mL, which, according the WHO definition, is defined as low-level viremia (LLV)^2,3^.

The incidence of LLV among PLWH exhibited an increasing trend from 1.5% in 1999–2001 to 28.4% in 2012^4^ , and it was still on the rise in recent years^5,6^. A study in the US reported that 46% of ART-experienced PLWH have experienced LLV, including both intermittent LLV (iLLV/blip) and persistent LLV (pLLV)^7^, while the incidence of LLV in China is about 10–30%^8–10^. LLV has been reported to be associated with various clinical poor outcomes such as HIV drug resistance and virological failure^5,11,12^. Several factors have been determined to be associated with the incidence of LLV by retrospective analysis. The clinical implication of LLV and its impact on clinical prognosis remain inconclusive^13,14^, but the ongoing process of HIV evolution and accumulation of drug resistance mutations (DRMs) during LLV is well recognized^15,16^. The incidence of DRMs in PLWH with LLV can reach as high as 10–40%, posing potential risk for subsequent virologic failure^16,17^.

At present, the source virus during LLV is hypothesized to be produced by two mechanisms: one is the persistent low-level viral production via cell cloning in sanctuary sites due to insufficient drug concentration^14,18^; and the other one is stimulated or clonally expanded CD4+ T cells that harbor integrated proviral DNA^19,20^. PLWH with LLV due to persistent low-level viral replication has a higher risk of developing DRMs. Risk factors for the development of LLV have been studied but remain inconclusive^8,12,17^. To understand the drug resistance profiles of HIV in LLV individuals in Zhengzhou City and provide data for the selection of appropriate subsequent ART regimens, we performed a detailed analysis of DRMs in PLWH with LLV admitted to our hospital from January 2022 to August 2023.

## Results

### Patient characteristics

During the study period, 120 (3.32%) out of 3616 PLWH were classified as having LLV with 57 (54.81%) as iLLV and 47 (45.19%) as pLLV. Among them, 104 (86.67%, 104/120) partial pol and full-length INT gene sequences were successfully obtained, of which 57 (54.81%) were from iLLV individuals and 47 (45.19%) were from pLLV. Therefore, 104 individuals were eligible for analysis in this study, with a median age of 45.5 years (IQR, 34–57). In all, male individuals accounted for 78.85% (82/104); 58.65% (61/104) of the individuals were married. The main route of transmission was heterosexual orientation, accounting for 40.38% (42/104), followed by men who have sex with men (MSM) (34.62%, 36/104), and plasmapheresis (17.31%, 18/104). In 83 individuals with baseline CD4+ T-cell count available, the median baseline CD4+ T-cell count was 216 cells/µL (min/max, 3/716). In 80 individuals with CD4+ T-cell count at LLV available, the median baseline CD4+ T-cell count was 374 cells/µL (min/max, 81/1826). In 48 individuals with baseline VL data, the median baseline VL was 45,744 (min/max, 150/2,498,565) copies/mL. The median VL at LLV was 96 (min/max, 53/983) copies/mL, detailed demographic characteristics of the PLWH are listed in Table 1.

**Table 1:** Characteristics of people living with HIV (PLWH) who presented with low-level viremia in Zhengzhou City, 2023.

	All (N = 104)		DRMs (N = 44)		NON-DR (N = 60)		*χ*^*2*^	*p*								
Sex, n (%)	104	100.00%	44	42.31%	60	57.69%	0.676	0.47								
Male	82	78.85%	33	40.24%	49	59.76%										
Female	22	21.15%	11	50.00%	11	50.00%										
Age at diagnosis, years, median, (IQR)	45.50 (34.00, 56.75)		44.5 (28.25, 56.75)		46.5 (36.25, 57.50)		2.272	0.135								
< 20 years, n (%)	1	0.96%	1	100.00%	0	0.00%	17.271	0.004								
20–29 years, n (%)	12	11.54%	11	91.67%	1	8.33%										
30–39 years, n (%)	27	25.96%	8	29.63%	19	70.37%										
40–49 years, n (%)	18	17.31%	5	27.78%	13	72.22%										
50–59 years, n (%)	26	25.00%	12	46.15%	14	53.85%										
≥ 60 years,n (%)	20	19.23%	7	35.00%	13	65.00%										
Marriage, n (%)																
Married	61	58.65%	22	36.07%	39	63.93%	2.355	0.159								
Single	43	41.35%	22	51.16%	21	48.84%										
Transmission category, n (%)																
HSX	42	40.38%	15	35.71%	27	64.29%	6.317	0.177								
MSM	36	34.62%	14	38.89%	22	61.11%										
PL	18	17.31%	10	55.56%	8	44.44%										
MTCT	3	2.88%	3	100.00%	0	0.00%										
Other	5	4.81%	2	40.00%	3	60.00%										
Occupation, n (%)	71	68.27%	31	43.66%	40	56.34%										
Farmers	16	15.38%	12	75.00%	4	25.00%	10.907	0.012								
Workers	44	42.31%	16	36.36%	28	63.64%										
Non-workers	10	9.62%	2	20.00%	8	80.00%										
Other/unknown	34	32.69%	14	41.18%	20	58.82%										
Education, n (%)	71	68.27%	31	43.66%	40	56.34%										
College below	43	41.35%	16	37.21%	27	62.79%	1.846	0.223								
College or above	28	26.92%	15	53.57%	13	46.43%										
Unknown	33	31.73%	13	39.39%	20	60.61%										
Baseline CD4+ T cell count (cells/μl), N (%)	83	79.81%	33	39.76%	50	60.24%										
CD4+, median (min/max)	216 (3/716)		283 (4/550)		171.5 (3/716)		76.737	0.36								
HIV baseline viral load (copies/ml plasma)	48	46.15%	21	43.75%	27	56.25%										
VL, median (min/max)	45,744 (150/2,498,565)		48,888 (150/2,132,462)		42,600 (445/2,498,565)		48	0.432								
CD4+ T cell count at LLV (cells/μl), N (%)	104	100.00%	44	42.31%	60	57.69%										
CD4+, median (min/max)	354 (59/1534)		383.5 (81/1100)		343.5 (59/1534)		0.155	0.877								
HIV viral load at LLV (copies/ml plasma)	104	100.00%	44	42.31%	60	57.69%										
VL, median (min/max)	96 (53/983)		163.5 (57/983)		83.5 (53/544)		31.659	0.001								

	RNA								DNA							
	All (N = 70)		DRMs (N = 30)		NON-DR (N = 40)		*χ*^2^	*p*	All (N = 34)		DRMs (N = 14)		NON-DR (N = 20)		*χ*^2^	*p*
Sex, n (%)	70	100.00%	30	42.86%	40	38.46%	0.728	0.299	34	100.00%	14	41.18%	20	58.82%	0.123	0.505
Male	59	84.29%	24	40.68%	35	59.32%			23	67.65%	9	39.13%	14	60.87%		
Female	11	15.71%	6	54.55%	5	45.45%			11	32.35%	5	45.45%	6	54.55%		
Age at diagnosis, years, median, (IQR)	44.0 (34.00, 57.25)		46.5 (28.00, 58.25)		44 (35.00, 55.25)				47.5 (34.75, 56.50)		41.5 (28.25, 55.00)		49.5 (41.00, 58.75)			
< 20 years, n (%)	1	1.43%	1	100.00%	0	0.00%	15.45	0.009	0	0.00%	0	0.00%	0	0.00%	4.55	0.337
20–29 years, n (%)	7	10.00%	7	100.00%	0	0.00%			5	14.71%	4	80.00%	1	20.00%		
30–39 years, n (%)	21	30.00%	5	23.81%	16	76.19%			6	17.65%	3	50.00%	3	50.00%		
40–49 years, n (%)	10	14.29%	3	30.00%	7	70.00%			8	23.53%	2	25.00%	6	75.00%		
50–59 years, n (%)	17	24.29%	9	52.94%	8	47.06%			9	26.47%	3	33.33%	6	66.67%		
≥ 60 years,n (%)	14	20.00%	5	35.71%	9	64.29%			6	17.65%	2	33.33%	4	66.67%		
Marriage, n (%)																
Married	44	62.86%	18	40.91%	26	59.09%	5.179	0.159	17	50.00%	4	23.53%	13	76.47%	6.096	0.107
Single	26	37.14%	12	46.15%	14	53.85%			17	50.00%	10	58.82%	7	41.18%		
Transmission category, n (%)																
HSX	30	42.86%	10	33.33%	20	66.67%	4.991	0.288	12	35.29%	5	41.67%	7	58.33%	5.591	0.232
MSM	24	34.29%	11	45.83%	13	54.17%			12	35.29%	3	25.00%	9	75.00%		
PL	11	15.71%	7	63.64%	4	36.36%			7	20.59%	3	42.86%	4	57.14%		
MTCT	1	1.43%	1	100.00%	0	0.00%			2	5.88%	2	100.00%	0	0.00%		
Other	4	5.71%	1	25.00%	3	75.00%			1	2.94%	1	100.00%	0	0.00%		
Occupation, n (%)	44	62.86%	20	45.45%	24	54.55%			26	76.47%	10	38.46%	16	61.54%		
Farmers	12	17.14%	10	83.33%	2	16.67%	9.575	0.008	4	11.76%	2	50.00%	2	50.00%	3.674	0.299
Workers	25	35.71%	8	32.00%	17	68.00%			19	55.88%	8	42.11%	11	57.89%		
Non-workers	7	10.00%	2	28.57%	5	71.43%			3	8.82%	0	0.00%	3	100.00%		
Other/unknown	26	37.14%	10	0.00%	16	0.00%			8	23.53%	4	50.00%	4	50.00%		
Education, n (%)	44	62.86%	20	0.00%	24	54.55%			27	79.41%	11	40.74%	16	59.26%		
College below	27	38.57%	12	44.44%	15	55.56%	0.29	0.555	16	47.06%	4	25.00%	12	75.00%	4.03	0.054
College or above	17	24.29%	8	47.06%	9	52.94%			11	32.35%	7	63.64%	4	36.36%		
Unknown	26	37.14%	10	38.46%	16	61.54%			7	20.59%	3	42.86%	4	57.14%		
Baseline CD4+ T cell count (cells/μl), N (%)	57	81.43%	23	40.35%	34	59.65%			26	76.47%	10	38.46%	16	61.54%		
CD4+, median (min/max)	216 (4/716)		232(4/550)		188(11/716)		57	0.364	272 (3/509)		331.5 (10/472)		76 (3/509)		23.888	0.468
HIV baseline viral load (copies/ml plasma)	31	44.29%	14	45.16%	17	54.84%			17	50.00%	7	41.18%	10	58.82%		
VL, median (min/max)	48,888 (167/2,498,565)		46,266 (445/2,498,565)		48,888 (167/2,132,462)		31	0.415	42,600 (150/1,000,000)		42,600 (4673/424,867)		46,070.5 (150/1,000,000)		17	0.386
CD4+ T cell count at LLV (cells/μl), N (%)	58	82.86%	28	48.28%	30	51.72%			34	100.00%	14	41.18%	20	58.82%		
CD4+, median (min/max)	343.5 (59/1534)		293.5 (81/972)		346.5 (59/1534)		0.781	0.438	402.5 (83/1100)		495 (83/1100)		321 (135/942)		1.587	0.122
HIV viral load at LLV (copies/ml plasma)	70	100.00%	30	42.86%	40	57.14%			34	100.00%	14	41.18%	20	58.82%		
VL, median (min/max)	97 (53/983)		189.5 (57/983)		79.5 (53/544)		21.173	0.004	94 (55/870)		124.5 (60/870)		89.0 (55/525)		10.302	0.058

Data are presented as n (%) or median (IQR); IQR, interquartile range; significance for differences was measured using Chi-squared test, Fisher’s Exact test, or Kruskal–Wallis test. HSX, heterosexual orientation; MSM, men who have sex with men; MTCT, mother-to-child transmition; PL, plasmapheresis; IDU, injection drug use; OTH, others, including patients whose risk factors were unknown or patients who did not provide information; TDRM, transmitted drug-resistance mutations; NRTI, nucleotide reverse transcriptase inhibitors; NNRTI, non-nucleoside reverse transcriptase inhibitors; PI, protease inhibitors; INSTI, integrase strand transfer inhibitors.

### Genetic characterization

**Figure 1:**
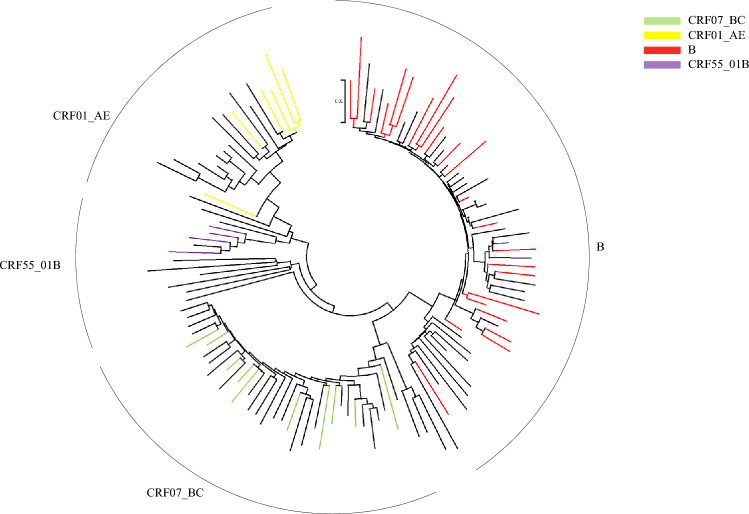
Phylogenetic tree based on partial pol sequence was constructed using Molecular Evolutionary Genetic Analysis (MEGA) software (version XI) based on neighbor-joining method and General Time Reversible model with 1000 bootstrap replicates. TDRMs of different subtypes are shown in different colors. Reference sequences (GenBank No. U51189, AF286226, AF286229, AF069670, AY945737, DQ207940, U21135, AF067155, JX574661, AF077336, AF061642, AF190127, AF082395, AJ249235, AF286236) were downloaded from the Los Alamos HIV Sequence Database (https://www.hiv.lanl.gov/).

Of the 104 partial pol gene sequences determined, 67.31% (70/104) were derived from HIV RNA. Subtyping analysis was performed by submitting sequences to the online REGA HIV-1 Subtyping Tool. According to the results, subtype B dominated (48.08%, 50/104), followed by CRF07_BC (31.73%, 33/104), CRF01_AE (15.38%, 16/104), CRF55_01B (3.85%, 4/104), and CRF08_BC (0.96%, 1/104). The distribution of subtypes was similar between the LLV samples genotyped by HIV-1 RNA based assay and HIV-1 proviral-DNA based assay (Table 2). Figure 1 shows the phylogenetic analysis with reference strains, including all confirmed subtypes. Based on the inferred phylogenetic tree, all determined subtypes clustered with reference strains, indicating consistent results and correct subtyping.

**Table 2:** Distribution and prevalence of DRMs among HIV-1 subtypes in PLWH with LLV in Zhengzhou City, 2023.

Subtype	Total						RNA						DNA					
	Patients		DRMs		*χ*^2^	*p*	Patients		DRMs		*χ*^2^	*p*	Patients		DRMs		*χ*^2^	*p*
	n	%	n	%			n	%	n	%			n	%	n	%		
B	50	48.08	24	48.00	8.952	0.062	35	50.00	17	24.29	5.553	0.135	15	44.12	7	20.59	4.653	0.325
CRF07_BC	33	31.73	10	30.30			25	35.71	7	10.00			8	23.53	3	8.82		
CRF01_AE	16	15.38	6	37.50			8	11.43	4	5.71			8	23.53	2	5.88		
CRF55_01B	4	3.85	4	100.00			2	2.86	2	2.86			2	5.88	2	5.88		
CRF08_BC	1	0.96	0	0.00			0	0.00	0	0.00			1	2.94	0	0.00		
Total	104	100.00	44	42.31			70	100.00	30	42.86			34	100.00	14	41.18		

Univariate logistic regression analysis was performed. CRF, circulating recombinant forms; DRMs, drug resistance mutations.

### Prevalence and distribution of DRMs

**Figure 2:**
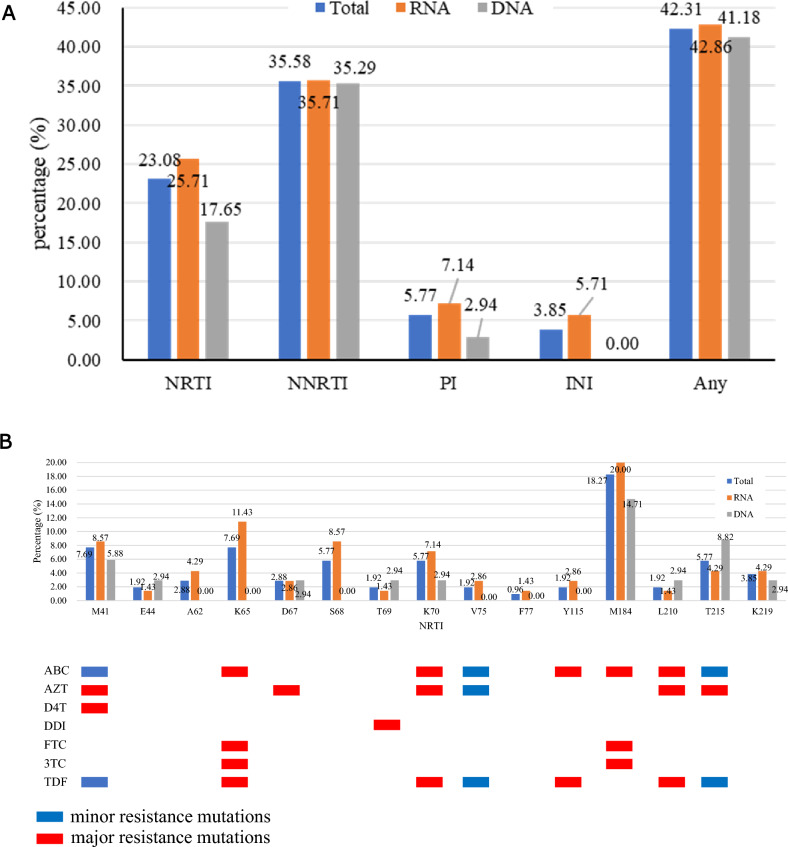

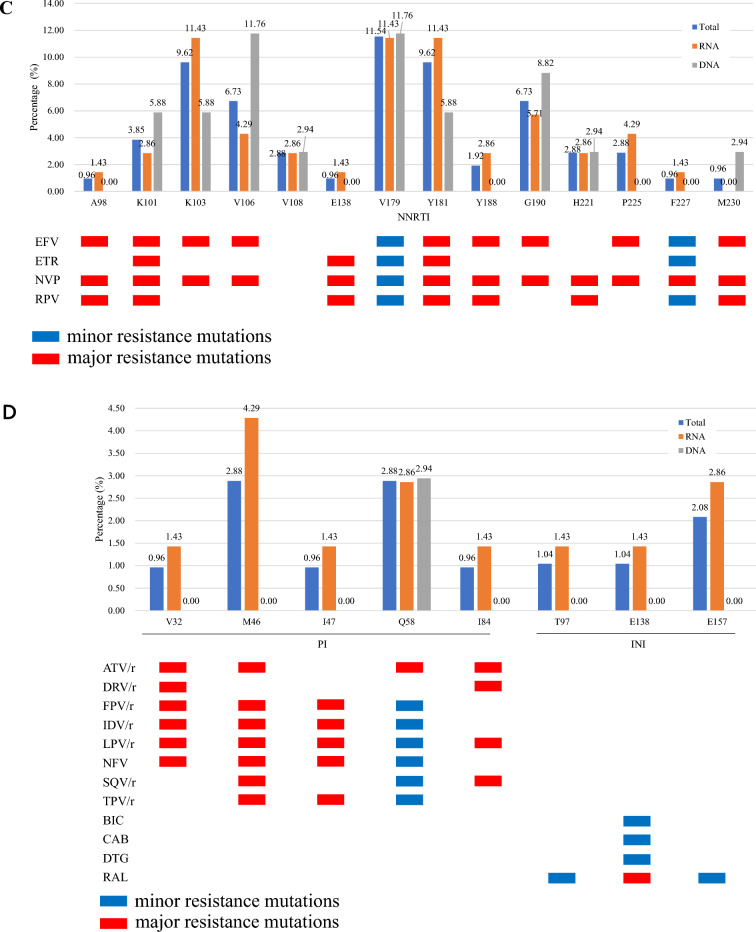
Distribution and prevalence of drug class-specific DRMs in PLWH with LLV in Zhengzhou City, 2023. (**A**) prevalence of DRMs for four drug classes (NNRTI, NRTI, PI, and INSTI); (**B**) Specific DRMs stratified by NRTI; (**C**) Specific DRMs stratified by NNRTI; (**D**) Specific DRMs stratified by PI and INSTI. The major and minor resistance mutations are highlighted in red and blue, respectively. NNRTI, non-nucleoside reverse transcriptase inhibitors; NRTI, nucleoside reverse transcriptase inhibitors; PI, protease inhibitors; INSTIs, integrase strand transfer inhibitors. Total: LLV samples genotypled by HIV-1 RNA or proviral-DNA; RNA: LLV samples genotypled by HIV-1 RNA; DNA: LLV samples genotypled by HIV-1 proviral-DNA. ABC, abacavir; AZT, zidovudine; FTC, emtricitabine; 3TC, lamivudine; TDF, tenofovir; DOR, doravirine; EFV, efavirenz; ETR, etravirine ; NVP, nevirapine; RPV, rilpivirine; DRV/r, darunavir/r; LPV/r, lopinavir/r; BIC, bictegravir; CAB, cabotegravir; DTG, dolutegravir; EVG, elvitegravir; RAL, raltegravir.

Among the 104 LLV individuals with available partial pol and full-length INT gene sequences, DRMs to any drug was detected in 42.31% (44/104). Resistance to NNRTIs was dominant (37/104, 35.58%), followed by NRTIs (24/104, 23.08%), PIs (6/104, 5.77%), and INSTIs (4/104, 3.85%) (Fig. 2A). V179D/E/L/VD (12, 11.54%), M184V/I (19, 18.27%), M46I and Q58E (3, 2.88%), and E157Q (2, 2.08%) were the most commonly observed mutations against NNRTIs, NRTIs, PIs, and INSTIs (Fig. 2, B-D). Further analysis indicated that 22 (21.15%) individuals were found with single-class, 17 (16.35%) with dual-class, and 5 (4.81%) with triple-class drug resistance (data not shown). When categorized by genotypes, DRMs was most observed in CRF55_01B (100.0%, 4/4), followed by B (48.00%, 24/50), CRF07_BC (30.30%, 10/33), and CRF01_AE (37.50%, 6/16) (Table 2).

**Figure 3:**
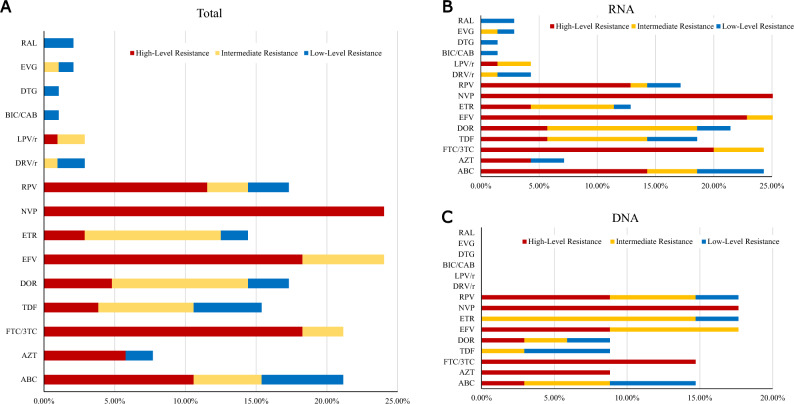
Predicted resistance to antiretroviral drugs among HIV-1 pol sequences with DRMs in PLWH with LLV in Zhengzhou City, 2023. (**A**) Different drug resistance levels of four classes of antiretroviral drugs predicted by the Stanford HIV Drug Resistance Database in LLV samples genotyped by HIV-1 RNA or proviral-DNA; (**B**) Different drug resistance levels of four classes of antiretroviral drugs predicted by the Stanford HIV Drug Resistance Database in LLV samples genotyped by HIV-1 RNA; (**C**) Different drug resistance levels of four classes of antiretroviral drugs predicted by the Stanford HIV Drug Resistance Database in LLV samples genotyped by HIV-1 proviral-DNA. ABC, abacavir; AZT, zidovudine; FTC, emtricitabine; 3TC, lamivudine; TDF, tenofovir; DOR, doravirine; EFV, efavirenz; ETR, etravirine ; NVP, nevirapine; RPV, rilpivirine; DRV/r, darunavir/r; LPV/r, lopinavir/r; BIC, bictegravir; CAB, cabotegravir; DTG, dolutegravir; EVG, elvitegravir; RAL, raltegravir.

We further analyzed each drug independently. NVP dominated in high-level, DOR and RPV dominated in medium-level, ABC dominated in low-level, and ETR dominated in potentially low-level of resistant drugs. As more than 90% of PLWH would choose the current free drug regimens for treatment, prevalence of DRMs against the available free ART drugs (ABC, AZT, 3TC and TDF of NRTIs, NVP and EFV of NNRTIs, and LPV/r of PIs) in China were analyzed. Our results indicated, the overall prevalence of DRMs to the seven free ART drugs was 36.54% (38/104), while DRMs that led to low- or higher-level resistance was 25.96% (27/104). These included low- or higher-level resistance to ABC, AZT, 3TC and TDF of the NRTIs in 21.15% (22/104), 7.69% (8/104), 21.15% (22/104), and 15.38% (16/104), respectively; to NVP and EFV of the NNRTIs in 24.04% (25/104) and 24.04% (25/104), respectively; and to LPV/r of the PIs in 2.88% (3/104). Detailed information about the DRMs-related drug resistance to ART regimens is shown in Fig. 3.

### Influencing factors of DRMs in LLV individuals

Univariate logistic regression analysis was used to analyze the influencing factors associated with DRMs in PLWH with LLV (Tables 3 and 4). According to our results, individuals of less than 30-years old were more likely to develop DRMs than other age groups (OR = 22.286, 95% CI = 2.379–208.789, P < 0.05). The genotyping success rates of PLWH with VL of 50–200, 201–400, 401–999 copies/mL groups were 85.14% (63/74), 84.21% (16/19), and 81.82% (9/11), respectively, and there was no statistical difference in genotyping success rate (data not shown). The incidence of DRMs in LLV individuals with VL between 401 and 999 copies/mL (OR = 9.375, 95% CI = 1.878–46.790, P < 0.05) and 201 and 400 copies/mL (OR = 2.865, 95% CI = 1.020–8.045, P < 0.05) was significantly higher than that between 50 and 200 copies/mL. The association analysis indicated that individuals with LLV receiving 2NRTIs + PI/r regimen when LLV (including both iLLV and pLLV) was detected were more likely to develop DRMs (OR = 19.500, 95% CI = 3.585–106.077, P < 0.05). Other factors such as sex, CD4+ T cell count during LLV, delay of ART initiation, ART duration, occupation, transmission category, education, and marriage status were also analyzed, but were not significantly associated with DRMs.

**Table 3:** Analysis of influencing factors of DRMs among PLWH with LLV in Zhengzhou City, 2023.

	Total	DRMs	OR (95% CI)	*P*
Sex, n (%)	104 (100%)	44 (42.31%)		
Male	82 (78.10%)	33 (40.24%)	0.673 (0.262–1.733)	0.412
Female	23 (21.90%)	11 (47.83%)	1	
Age at diagnosis, years, median, (IQR)	45.50 (34.00, 56.75)	44.5 (28.25, 56.75)		
< 30 years, n (%)	13 (12.50%)	12 (92.31%)	22.286 (2.379–208.789)	0.007
30–39 years, n (%)	27 (25.96%)	8 (29.63%)	0.782 (0.227–2.690)	0.696
40–49 years, n (%)	18 (17.31%)	5 (27.78%)	0.714 (0.179–2.843)	0.633
50–59 years, n (%)	26 (25.00%)	12 (46.15%)	1.592 (0.480–5.282)	0.447
≥ 60 years, n (%)	20 (19.23%)	7 (35.00%)	1	
VL at LLV, median (IQR), copies/ml plasma	96 (65.00, 230.25)	163.5 (73.25, 374.75)		
50–200	74 (71.15%)	24 (32.43%)	1	
201–400	19 (18.27%)	11(57.89%)	2.865 (1.020–8.045)	0.046
401–999	11 (10.58%)	9 (81.82%)	9.375 (1.878–46.790)	0.006
CD4+ T cell count at LLV, median (IQR), cells/µL	354 (203.50–547.00)	416.6 (207.00–536.00)		
< 100	6 (5.77%)	3 (50.00%)	1.300 (0.245–6.903)	0.758
100–250	29 (27.88%)	11 (37.93%)	0.794 (0.327–1.931)	0.612
≥ 250	69 (66.35%)	30 (43.48%)	1	
Delay of ART initiation (IQR), months	0 (0–2)	0.5(0–3)		
0	54 (20.19%)	21 (38.89%)	1.485 (0.345–6.387)	0.595
0.1–1	21 (7.69%)	8 (38.10%)	1.436 (0.286–7.212)	0.66
1.1–6	12 (7.69%)	8 (66.67%)	4.667 (0.765–28.466)	0.095
6.1–12	7 (3.85%)	4 (57.14%)	3.111 (0.414–23.393)	0.27
> 12	10 (2.88%)	3 (30.00%)	1	
Time since ART initiation, median (IQR), years	5 (2.5–8.0)	5 (3–9.75)		
≤ 1	13 (5.77%)	6 (46.15%)	0.798 (0.218–2.925)	0.734
1–5	52 (12.50%)	13 (25.00%)	0.802 (0.237–2.712)	0.723
≥ 5	54 (21.15%)	22 (40.74%)	1	
Initial ART regimen				
2NRTI + NNRTI	83 (79.81%)	37 (44.58%)	1.688 (0.534–5.334)	0.372
2NRTI + PI/r	5 (4.81%)	2 (40.00%)	1.467 (0.184–11.718)	0.718
2NRTI + INSTI	16 (15.38%)	5 (31.25%)	1	
ART regimen when LLV was detected				
2NRTI + NNRTI	61 (58.65%)	24 (39.34%)	2.625 (0.986–6.988)	0.053
2NRTI + PI/r	14 (13.46%)	12 (85.71%)	19.500 (3.585–106.077)	0.001
2NRTI + INSTI	29 (27.88%)	8 (27.59%)	1	
LLV type				
iLLV	57 (54.81%)	22 (38.60%)	1	
pLLV	47 (45.19%)	22 (46.81%)	1.400 (0.640–3.062)	0.088

**Table 4:** Analysis of influencing factors of DRMs among PLWH between iLLV and pLLV in Zhengzhou City, 2023.

Characteristics	pLLV				iLLV			
	Total	DRMs	OR (95% CI)	*P*	Total	DRMs	OR (95% CI)	*P*
Sex, n (%)	47 (100.00%)	22 (46.81%)			57 (100.00%)	22 (38.60%)		
Male	40 (85.11%)	19 (47.50%)	1.206 (0.239–6.099)	0.821	42 (73.68%)	14 (33.33%)	0.438 (0.132–1.453)	0.177
Female	7 (14.89%)	3 (42.86%)	1		15 (26.32%)	8 (53.33%)	1	
Age at diagnosis, years, median, (IQR)								
< 30 years, n (%)	8 (17.02%)	7 (87.50%)	8.750 (0.737–103.824)	0.086	5 (8.77%)	5 (100.00%)		
30–39 years, n (%)	13 (27.66%)	3 (23.08%)	0.375 (0.059–2.366)	0.397	14 (24.56%)	5 (35.71%)		
40–49 years, n (%)	10 (21.28%)	4 (40.00%)	0.833 (0.134–5.167)	0.845	8 (14.04%)	1 (12.50%)		
50–59 years, n (%)	7 (14.89%)	4 (57.14%)	1.667 (0.227–12.221)	0.615	19 (33.33%)	8 (42.11%)		
≥ 60 years, n (%)	9 (19.15%)	4 (44.44%)	1		11 (19.30%)	3 (27.27%)	1	
VL at LLV (copies/mL)								
50–200	34 (72.34%)	12 (35.29%)			40 (70.18%)	12 (30.00%)	0.429 (0.054–3.408)	0.423
201–400	6 (12.77%)	3 (50.00%)			13 (22.81%)	8 (61.54%)	1.600 (0.168–15.273)	0.683
401–1000	7 (14.89%)	7 (100.00%)			4 (7.02%)	2 (50.00%)	1	
CD4+ T cell count at LLV, median (IQR), cells/µL								
< 100	2 (4.26%)	1 (50.00%)	0.375 (0.020–6.997)	0.511	1 (1.75%)	1 (100.00%)		
100–250	10 (21.28%)	3 (30.00%)	0.161 (0.031–0.834)	0.03	15 (26.32%)	7 (46.67%)		
≥ 250	22 (46.81%)	16 (72.73%)	1		30 (52.63%)	12 (40.00%)		
Log (baseline VL)								
< 3	3 (6.38%)	0 (0.00%)			3 (5.26%)	1 (33.33%)	1.500 (0.055–40.633)	0.81
3–5	15 (31.91%)	8 (53.33%)			12 (21.05%)	5 (41.67%)	2.143 (0.169–27.103)	0.556
≥ 5	11 (23.40%)	6 (54.55%)			4 (7.02%)	1 (25.00%)	1	
Baseline CD4+ T cell count median (IQR), cells/µL								
< 100	10 (21.28%)	3 (30.00%)	0.273 (0.052–1.422)	0.123	14 (24.56%)	3 (21.43%)	0.273 (0.056–1.319)	0.106
100–250	12 (25.53%)	5 (41.67%)	0.455 (0.103–2.013)	0.299	11 (19.30%)	2 (18.18%)	0.222 (0.037–1.330)	0.099
≥ 250	18 (38.30%)	11 (61.11%)	1		18 (21.58%)	9 (50.00%)	1	
Delay of ART initiation (IQR), months								
0	22 (46.81%)	10 (45.45%)			32 (56.14%)	11 (34.38%)		
0.1–1	9 (19.15%)	2 (22.22%)			12 (21.05%)	6 (50.00%)		
1.1–6	6 (12.77%)	4 (66.67%)			6 (10.53%)	4 (66.67%)		
6.1–12	3 (6.38%)	3 (100.00%)			4 (7.02%)	1 (25.00%)		
> 12	7 (14.89%)	3 (42.86%)			3 (5.26%)	0 (0.00%)		
Time since ART initiation, median (IQR), years								
≤ 1	6 (12.77%)	3 (50.00%)	1.400 (0.233–8.421)	0.713	7 (12.28%)	3 (42.86%)	1.125 (0.213–5.950)	0.89
1–5	14 (29.79%)	8 (57.14%)	1.867 (0.492–7.085)	0.359	18 (31.58%)	5 (27.78%)	0.577 (0.163–2.042)	0.394
≥ 5	24 (51.06%)	10 (41.67%)	1		30 (52.63%)	12 (40.00%)	1	
Initial ART regimen								
NRTI + NNRTI	31 (65.96%)	18 (58.06%)			45 (78.95%)	15 (33.33%)	0.750 (0.113–4.892)	0.766
NRTI + PI	2 (4.26%)	0 (0.00%)			3 (5.26%)	2 (66.67%)	3.000 (0.150–59.890)	0.472
NRTI + INSTI	11 (23.40%)	3 (27.27%)			5 (8.78%)	2 (40.00%)	1	
ART regimen when LLV was detected								
NRTI + NNRTI	20 (42.55%)	13 (65.00%)	6.500 (1.537–27.486)	0.011	27 (47.37%)	8 (29.63%)	1.263 (0.311–5.128)	0.744
NRTI + PI	5 (10.64%)	4 (80.00%)	14.000 (1.200–163.367)	0.035	9 (15.79%)	8 (88.89%)	24.000 (2.251–255.938)	0.008
NRTI + INSTI	18 (38.30%)	4 (22.22%)	1		16 (28.07%)	4 (25.00%)	1	

## Discussion

In this study we investigated the prevalence of, and DRMs associated with LLV in PLWH in Zhengzhou City. Due to technical problems, most RNA-based genotyping assays require the HIV VL to be above 1000 copies/mL, which is also an important reason for the limited data on DRMs in LLV individuals. Therefore, although genotypic drug resistance assay based on HIV proviral DNA is not routinely used for clinical monitoring, it still can be very useful in PLWH when plasma sequencing is not successful^21^. Our results showed that among 3616 ART-experienced PLWH in a long-term follow-up cohort from Jan 2022 to Aug 2023 in Zhengzhou City, 120 were identified as having LLV, giving a prevalence of 3.32%. Of these LLV individuals, males accounted for 78.10% (82/104). When stratified by age, LLV incidence among PLWH was higher in the age group of 30–39 (25.96%, 27/104) and 50–59 (25.00%, 26/104), and the incidence of PLWH over 50-year old was 44.23% (46/104), which is consistent with other studies in China^12,22^. Notably, due to historical reasons in the 1990s and a large proportion of such patients, plasmapheresis accounted for 17.31% (18/104) of total LLV individuals.

Our data showed that the incidence of DRM increases with the increase of VL in LLV individuals, especially in pLLV individuals (Tables 3 and 4). Both iLLV and pLLV during ART have been reported to be associated with increased risk of subsequent virologic failure^13^. A study in Botswana indicated that a single LLV during ART can strongly predict the risk of future virologic failure^23^. A study in the US revealed that the magnitude of LLV was the primary driver of evolution rate at both DRM and non-DRM sites, and higher VL was associated with the development of DRM^24^. These findings may provide applicable insights to the management of LLV individuals during ART.

Our results indicated that subtype B dominated (48.08%, 50/104), followed by CRF07_BC (31.73%, 33/104), CRF01_AE (15.38%, 16/104), CRF55_01B (3.85%, 4/104), and CRF08_BC (0.96%, 1/104). This is different from our previous studies in newly diagnosed PLWH in Henan Province in which CRF07_BC dominated, followed by CRF01_AE and B^25,26^, while in treatment experienced PLWH with viremia subtype B dominated, followed by CRF01_AE and CRF07_BC^27^, indicating specific genotype distribution characteristics in LLV individuals. It is worth noting that the genotype distribution in LLV individuals in Zhengzhou City is also different from those in Jiangsu and Guangdong Provinces^22,28^, suggesting regional differences.

42.31% (44/104) of LLV individuals in our study developed DRMs, lower than the overall resistance prevalence of 74% in Italy^4^. 25.96% had low- or higher-level resistance to the seven free ART drugs (ABC, AZT, 3TC, TDF, EFV, NVP, and LPV/r), showing a similar trends to studies among LLV individuals in Guangdong and Jiangsu Provinces, China^22,28^. It should be noted that when different definition of LLV was adopted, the prevalence of DRMs in LLV individuals can vary widely from 17 to 75%^29,30^ in different countries or regions. The incidence of DRMs was 23.08% (24/104), 35.58% (37/104), 5.77% (6/104), and 3.85% (4/104) for NRTIs, NNRTIs, PIs, and INSTIs, respectively (Fig. 2A). M184V/I (19, 18.27%), V179D/E/L/VD (12, 11.54%), M46I and Q58E (3, 2.88%), and E157Q (2, 2.08%) were the most commonly detected mutations in NRTIs, NNRTIs, PIs, and INSTIs (Fig. 2, B-D). In addition, K103N/S (10, 9.62%) and Y181C/I (10, 9.62%) were also commonly observed, which were comparable to other studies^12,16,28,31^. M184V/I, mainly selected by FTC or 3TC, has strong effect on viral replication capacity, though 68% can revert back to wild type^32^. V179D was a mutation related to the potential low-level resistance to EFV and NVP^33^. Q58E/QE is a common accessory DRM mutation associated with PI in many studies^34^. E157Q is accounted for potential resistance to RAL and EVG^34^. K103 plus M184 mutations can even lead to the antiviral failure of ART regimens comprising FTC, 3TC, EFV, or NVP^35^. High prevalence of DRMs in LLV individuals, and the detection of DRMs in all ART drug classes suggest the importance of monitoring DRMs.

Our results are consistent with reports in that LLV individuals using PI-based regimens for initial ART are less likely to develop DRMs than those using NNRTI-based regimens, while individuals with the VL between 401 and 999 copies /mL (OR = 9.375, 95% CI = 1.878–46.790, P < 0.05) and 201 and 400 copies /mL (OR = 2.865, 95% CI = 1.020–8.045, P < 0.05) were more likely to develop DRMs than that between 50 and 200 copies/mL^16,28^. It is worth noting that DRMs are more likely to be detected in individuals receiving PI-based regimens when LLV is detected (OR = 19.500, 95% CI = 3.585–106.077, P < 0.05), which most possibly is due to previous failure to first-line antiretroviral regimens consisting of NNRTIs and NRTIs as PIs-associated DRMs are rarely detected.

Our study has several limitations. First, the small sample size could have limited the power of the statistical and correlation analysis. Second, individuals in the VL group of 50–200 copies/mL accounted for 71.15%, which was significantly higher, and may bring bias to subsequent analysis. Third, influencing factors could not be accurately assessed for the lack of some clinical information in some individuals, such as occupation, education, CD4 + T cell count, and adherence. Fourth, some of the results were interpreted using proviral DNA-based genotyping, concordance between proviral DNA and RNA genotyping needs to be further improved. Nevertheless, our data indicated that the prevalence of DRMs in ART-experienced PLWH with LLV is high in Zhengzhou City. Implementation of Genotypic drug resistance testing for PLWH with LLV can facilitate early identification of DRMs and provision of effective treatment.

## Materials and methods

### Ethical statement

This study was approved by the Institutional Ethics Committee of The Sixth People’s Hospital of Zhengzhou, China (IEC-KY-2022-005-2) and performed in compliance with all relevant ethical regulations such as the Declaration of Helsinki (2008). Signed informed consent was obtained from each individual before the collection of blood samples.

### Study population

There are approximately 4000 PLWH in Zhengzhou City and each year they come to the Sixth People’s Hospital of Zhengzhou for annual monitoring of their VL and CD4+ T-cell count. PLWH who had experienced ART for at least 6 months when visiting the Sixth People’s Hospital of Zhengzhou from January 2022 to August 2023 and exhibited a VL greater than 50 copies/mL and less than 1,000 copies/mL at one time point (iLLV/blips) and/or two consecutive time points (pLLV) with previously undetected VL (< 50 copies/mL) were included in this analysis. Their plasma samples during LLV were collected for genotypic drug resistance assay. Demographic data and medical records, including HIV-VL, CD4+ T-cell count, and transmission route, were collected. Patients’ informed consent of each participant was obtained before sample collection.

### Nucleic acid purification

DNA extraction: whole blood samples were collected and centrifuged at low speed to obtain the buffy coat, which were then used for HIV-1 DNA extraction following the instructions of a Blood Genomic DNA Extraction kit (CWBio, Jiangsu, China) as per the manufacturer’s instructions.

RNA extraction: plasma samples (1–5 mL) were concentrated at 28,000 g for 30 min at 4 °C by ultracentrifugation to enrich HIV. The pellet was resuspended in phosphate buffered saline (0.01 M, pH 7.2) and then used for RNA extraction using an RNA extraction kit (Liferiver, Shanghai, China) by following the manufacturer’s instructions.

### Genotypic drug resistance testing

An In-house genotypic drug resistance testing was performed as described previously^27^. Briefly, HIV-1 RNA was used to amplify HIV-1 partial pol gene sequence and full-length integrase (INT) gene sequence using reverse transcription and nested-PCR, or alternatively HIV proviral DNA was used when HIV RNA based amplification failed. The HIV-1 *pol* and INT gene was reverse transcribed using the specific primer R5073 and a RevertAid First Strand cDNA Synthesis Kit (Thermo Fisher Scientific, MA, USA). The target sequence of *pol* gene (approximately 1300 bp) was amplified by nested PCR using LA Taq™ Version 2.0 (Takara, Shiga, Japan) with two primer sets (PRO-F1 and RT-R1 for first-round PCR, and PRO-F2 and RT-R2 for second-round PCR). Each amplified fragment was sequenced with two primer sets (CF1, CF3, CR2, and CR4 as the primary sequencing primer set; CF2, CF4, CR1, and CR3 as the backup sequencing primer set) (Supplementary Table 1). The target sequence of INT gene (approximately 700 bp) was amplified by nested PCR using two primer sets (F4181 and R5073 for first-round PCR, and F4379 and R5057 for second-round PCR). Each amplified fragment was sequenced using two primer sets (CF5 and CR5 as the primary sequencing primer set, and CF6 and R5057 as backup sequencing primer set) (Supplementary Table 1). The positive PCR products were purified, sequenced using Sanger sequencing, and then submitted to the regularly updated Stanford HIV-1 drug resistance database (http://hivdb.stanford.edu/) for DRMs and antiretroviral susceptibility analysis. Polymorphic mutations and polymorphic accessory mutations in combination with other DRMs might contribute to reduced susceptibility of certain antiretroviral drugs, and they are thus included in our analysis.

### Subtyping and phylogenetic analysis

Subtyping and phylogenetic analyses were performed as described previously. Briefly, subtypes of HIV-1 isolates were determined by REGA HIV-1 Subtyping Tool (http://dbpartners.stanford.edu:8080/RegaSubtyping/stanford-hiv/typingtool/) based on the partial pol region, which was further confirmed by phylogenetic analysis. The phylogenetic analysis was performed using Molecular Evolutionary Genetic Analysis (MEGA) software (version XI) based on the Maximum Likelihood method and General Time Reversible model. Tree topology was tested by bootstrap analysis with 1000 replicates. Reference sequences included in the ML tree (GenBank No. U51189, AF286226, AF286229, AF069670, AY945737, DQ207940, U21135, AF067155, JX574661, AF077336, AF061642, AF190127, AF082395, AJ249235, AF286236) were downloaded from the Los Alamos HIV Sequence Database (https://www.hiv.lanl.gov/).

### Statistical analysis

Statistical analysis was carried out using the SPSS statistics program for Windows (IBM SPSS Statistics, version 21). Continuous variables were expressed as mean ± standard deviation or a median with its interquantile range (IQR). Categorical variables were expressed as numbers or percentages. The differences between or among groups were analyzed by Student’s t-test or Chi-squared test; a P < 0.05 was considered to be statistically significant.

### Supplementary Information

Supplementary Information.

Supplementary Tables.

## Data Availability

*Accession numbers* The sequences determined in this study were deposited in the GenBank database under the accession numbers PP111491-PP111594.
