# Supplementary material for: Characteristics of drug resistance mutations in ART-experienced HIV-1 patients with low-level viremia in Zhengzhou City, China
Source: Sci Rep. 2024 May 9;14:10620. doi: 10.1038/s41598-024-60965-z (PMC11082154; doi:10.1038/s41598-024-60965-z)
Supplement: Supplementary file 2 — Supplementary Tables. [file 41598_2024_60965_MOESM2_ESM.pptx]

## Slide 1
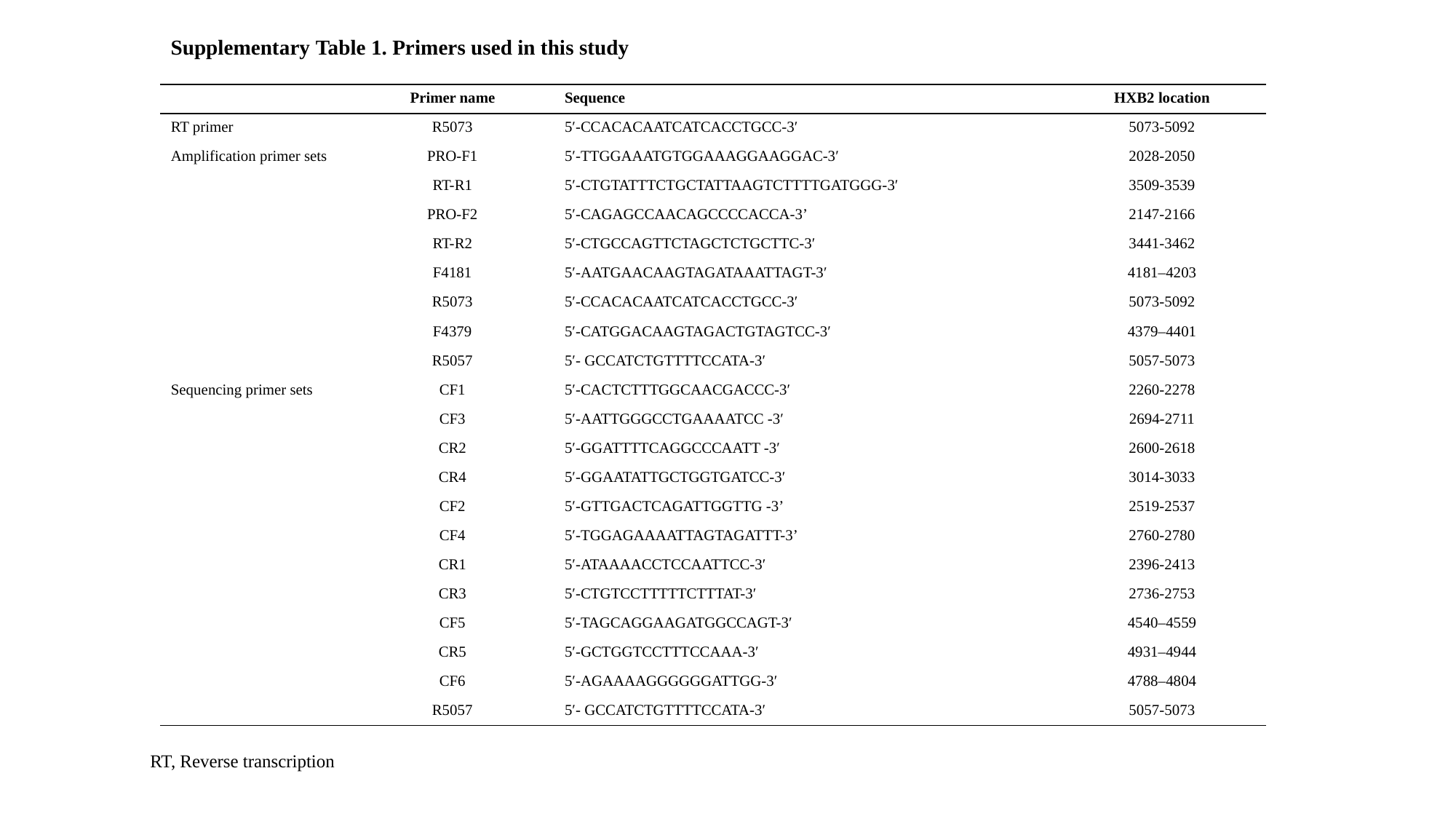

Supplementary Table 1. Primers used in this study
| | Primer name | Sequence | HXB2 location |
| --- | --- | --- | --- |
| RT primer | R5073 | 5′-CCACACAATCATCACCTGCC-3′ | 5073-5092 |
| Amplification primer sets | PRO-F1 | 5′-TTGGAAATGTGGAAAGGAAGGAC-3′ | 2028-2050 |
| | RT-R1 | 5′-CTGTATTTCTGCTATTAAGTCTTTTGATGGG-3′ | 3509-3539 |
| | PRO-F2 | 5′-CAGAGCCAACAGCCCCACCA-3’ | 2147-2166 |
| | RT-R2 | 5′-CTGCCAGTTCTAGCTCTGCTTC-3′ | 3441-3462 |
| | F4181 | 5′-AATGAACAAGTAGATAAATTAGT-3′ | 4181–4203 |
| | R5073 | 5′-CCACACAATCATCACCTGCC-3′ | 5073-5092 |
| | F4379 | 5′-CATGGACAAGTAGACTGTAGTCC-3′ | 4379–4401 |
| | R5057 | 5′- GCCATCTGTTTTCCATA-3′ | 5057-5073 |
| Sequencing primer sets | CF1 | 5′-CACTCTTTGGCAACGACCC-3′ | 2260-2278 |
| | CF3 | 5′-AATTGGGCCTGAAAATCC -3′ | 2694-2711 |
| | CR2 | 5′-GGATTTTCAGGCCCAATT -3′ | 2600-2618 |
| | CR4 | 5′-GGAATATTGCTGGTGATCC-3′ | 3014-3033 |
| | CF2 | 5′-GTTGACTCAGATTGGTTG -3’ | 2519-2537 |
| | CF4 | 5′-TGGAGAAAATTAGTAGATTT-3’ | 2760-2780 |
| | CR1 | 5′-ATAAAACCTCCAATTCC-3′ | 2396-2413 |
| | CR3 | 5′-CTGTCCTTTTTCTTTAT-3′ | 2736-2753 |
| | CF5 | 5′-TAGCAGGAAGATGGCCAGT-3′ | 4540–4559 |
| | CR5 | 5′-GCTGGTCCTTTCCAAA-3′ | 4931–4944 |
| | CF6 | 5′-AGAAAAGGGGGGATTGG-3′ | 4788–4804 |
| | R5057 | 5′- GCCATCTGTTTTCCATA-3′ | 5057-5073 |
RT, Reverse transcription

## Slide 2
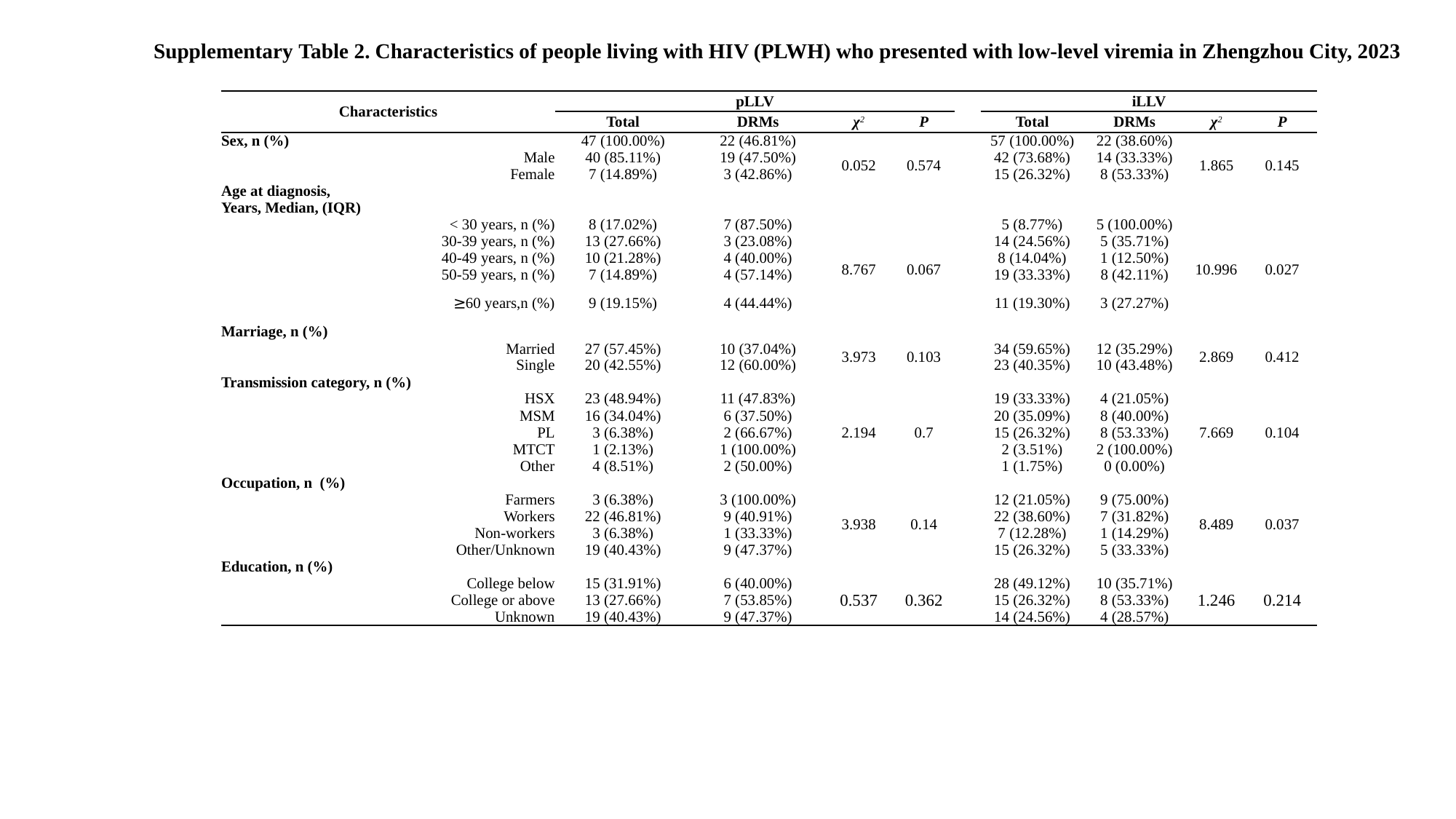

Supplementary Table 2. Characteristics of people living with HIV (PLWH) who presented with low-level viremia in Zhengzhou City, 2023
| Characteristics | pLLV | | | | | iLLV | | | |
| --- | --- | --- | --- | --- | --- | --- | --- | --- | --- |
| | Total | DRMs | χ2 | P | | Total | DRMs | χ2 | P |
| Sex, n (%) | 47 (100.00%) | 22 (46.81%) | | | | 57 (100.00%) | 22 (38.60%) | | |
| Male | 40 (85.11%) | 19 (47.50%) | 0.052 | 0.574 | | 42 (73.68%) | 14 (33.33%) | 1.865 | 0.145 |
| Female | 7 (14.89%) | 3 (42.86%) | | | | 15 (26.32%) | 8 (53.33%) | | |
| Age at diagnosis, Years, Median, (IQR) | | | | | | | | | |
| < 30 years, n (%) | 8 (17.02%) | 7 (87.50%) | 8.767 | 0.067 | | 5 (8.77%) | 5 (100.00%) | 10.996 | 0.027 |
| 30-39 years, n (%) | 13 (27.66%) | 3 (23.08%) | | | | 14 (24.56%) | 5 (35.71%) | | |
| 40-49 years, n (%) | 10 (21.28%) | 4 (40.00%) | | | | 8 (14.04%) | 1 (12.50%) | | |
| 50-59 years, n (%) | 7 (14.89%) | 4 (57.14%) | | | | 19 (33.33%) | 8 (42.11%) | | |
| ≥60 years,n (%) | 9 (19.15%) | 4 (44.44%) | | | | 11 (19.30%) | 3 (27.27%) | | |
| Marriage, n (%) | | | | | | | | | |
| Married | 27 (57.45%) | 10 (37.04%) | 3.973 | 0.103 | | 34 (59.65%) | 12 (35.29%) | 2.869 | 0.412 |
| Single | 20 (42.55%) | 12 (60.00%) | | | | 23 (40.35%) | 10 (43.48%) | | |
| Transmission category, n (%) | | | | | | | | | |
| HSX | 23 (48.94%) | 11 (47.83%) | 2.194 | 0.7 | | 19 (33.33%) | 4 (21.05%) | 7.669 | 0.104 |
| MSM | 16 (34.04%) | 6 (37.50%) | | | | 20 (35.09%) | 8 (40.00%) | | |
| PL | 3 (6.38%) | 2 (66.67%) | | | | 15 (26.32%) | 8 (53.33%) | | |
| MTCT | 1 (2.13%) | 1 (100.00%) | | | | 2 (3.51%) | 2 (100.00%) | | |
| Other | 4 (8.51%) | 2 (50.00%) | | | | 1 (1.75%) | 0 (0.00%) | | |
| Occupation, n (%) | | | | | | | | | |
| Farmers | 3 (6.38%) | 3 (100.00%) | 3.938 | 0.14 | | 12 (21.05%) | 9 (75.00%) | 8.489 | 0.037 |
| Workers | 22 (46.81%) | 9 (40.91%) | | | | 22 (38.60%) | 7 (31.82%) | | |
| Non-workers | 3 (6.38%) | 1 (33.33%) | | | | 7 (12.28%) | 1 (14.29%) | | |
| Other/Unknown | 19 (40.43%) | 9 (47.37%) | | | | 15 (26.32%) | 5 (33.33%) | | |
| Education, n (%) | | | | | | | | | |
| College below | 15 (31.91%) | 6 (40.00%) | 0.537 | 0.362 | | 28 (49.12%) | 10 (35.71%) | 1.246 | 0.214 |
| College or above | 13 (27.66%) | 7 (53.85%) | | | | 15 (26.32%) | 8 (53.33%) | | |
| Unknown | 19 (40.43%) | 9 (47.37%) | | | | 14 (24.56%) | 4 (28.57%) | | |

## Slide 3
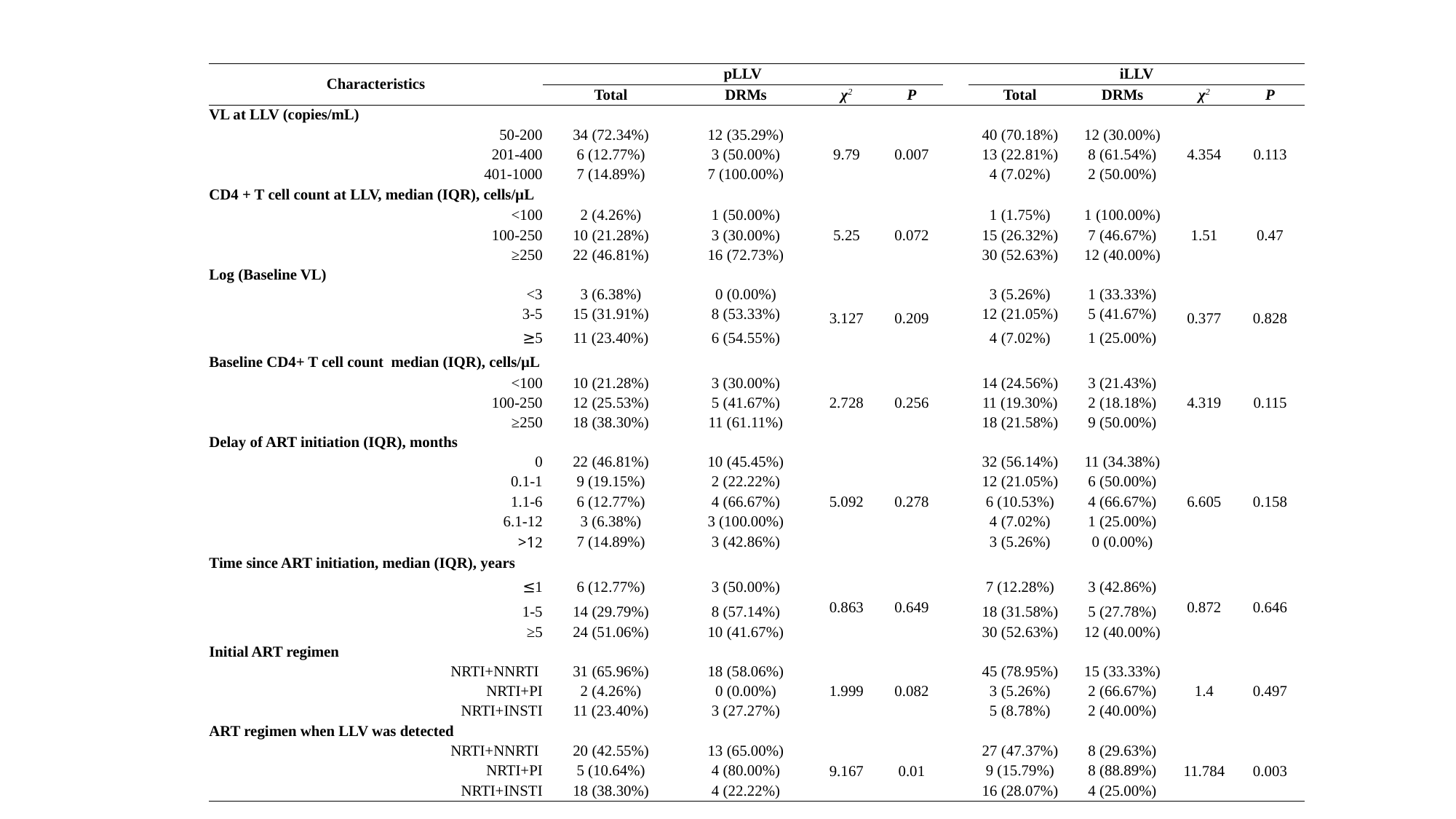

| Characteristics | pLLV | | | | | iLLV | | | |
| --- | --- | --- | --- | --- | --- | --- | --- | --- | --- |
| | Total | DRMs | χ2 | P | | Total | DRMs | χ2 | P |
| VL at LLV (copies/mL) | | | | | | | | | |
| 50-200 | 34 (72.34%) | 12 (35.29%) | 9.79 | 0.007 | | 40 (70.18%) | 12 (30.00%) | 4.354 | 0.113 |
| 201-400 | 6 (12.77%) | 3 (50.00%) | | | | 13 (22.81%) | 8 (61.54%) | | |
| 401-1000 | 7 (14.89%) | 7 (100.00%) | | | | 4 (7.02%) | 2 (50.00%) | | |
| CD4 + T cell count at LLV, median (IQR), cells/µL | | | | | | | | | |
| <100 | 2 (4.26%) | 1 (50.00%) | 5.25 | 0.072 | | 1 (1.75%) | 1 (100.00%) | 1.51 | 0.47 |
| 100-250 | 10 (21.28%) | 3 (30.00%) | | | | 15 (26.32%) | 7 (46.67%) | | |
| ≥250 | 22 (46.81%) | 16 (72.73%) | | | | 30 (52.63%) | 12 (40.00%) | | |
| Log (Baseline VL) | | | | | | | | | |
| <3 | 3 (6.38%) | 0 (0.00%) | 3.127 | 0.209 | | 3 (5.26%) | 1 (33.33%) | 0.377 | 0.828 |
| 3-5 | 15 (31.91%) | 8 (53.33%) | | | | 12 (21.05%) | 5 (41.67%) | | |
| ≥5 | 11 (23.40%) | 6 (54.55%) | | | | 4 (7.02%) | 1 (25.00%) | | |
| Baseline CD4+ T cell count median (IQR), cells/µL | | | | | | | | | |
| <100 | 10 (21.28%) | 3 (30.00%) | 2.728 | 0.256 | | 14 (24.56%) | 3 (21.43%) | 4.319 | 0.115 |
| 100-250 | 12 (25.53%) | 5 (41.67%) | | | | 11 (19.30%) | 2 (18.18%) | | |
| ≥250 | 18 (38.30%) | 11 (61.11%) | | | | 18 (21.58%) | 9 (50.00%) | | |
| Delay of ART initiation (IQR), months | | | | | | | | | |
| 0 | 22 (46.81%) | 10 (45.45%) | 5.092 | 0.278 | | 32 (56.14%) | 11 (34.38%) | 6.605 | 0.158 |
| 0.1-1 | 9 (19.15%) | 2 (22.22%) | | | | 12 (21.05%) | 6 (50.00%) | | |
| 1.1-6 | 6 (12.77%) | 4 (66.67%) | | | | 6 (10.53%) | 4 (66.67%) | | |
| 6.1-12 | 3 (6.38%) | 3 (100.00%) | | | | 4 (7.02%) | 1 (25.00%) | | |
| >12 | 7 (14.89%) | 3 (42.86%) | | | | 3 (5.26%) | 0 (0.00%) | | |
| Time since ART initiation, median (IQR), years | | | | | | | | | |
| ≤1 | 6 (12.77%) | 3 (50.00%) | 0.863 | 0.649 | | 7 (12.28%) | 3 (42.86%) | 0.872 | 0.646 |
| 1-5 | 14 (29.79%) | 8 (57.14%) | | | | 18 (31.58%) | 5 (27.78%) | | |
| ≥5 | 24 (51.06%) | 10 (41.67%) | | | | 30 (52.63%) | 12 (40.00%) | | |
| Initial ART regimen | | | | | | | | | |
| NRTI+NNRTI | 31 (65.96%) | 18 (58.06%) | 1.999 | 0.082 | | 45 (78.95%) | 15 (33.33%) | 1.4 | 0.497 |
| NRTI+PI | 2 (4.26%) | 0 (0.00%) | | | | 3 (5.26%) | 2 (66.67%) | | |
| NRTI+INSTI | 11 (23.40%) | 3 (27.27%) | | | | 5 (8.78%) | 2 (40.00%) | | |
| ART regimen when LLV was detected | | | | | | | | | |
| NRTI+NNRTI | 20 (42.55%) | 13 (65.00%) | 9.167 | 0.01 | | 27 (47.37%) | 8 (29.63%) | 11.784 | 0.003 |
| NRTI+PI | 5 (10.64%) | 4 (80.00%) | | | | 9 (15.79%) | 8 (88.89%) | | |
| NRTI+INSTI | 18 (38.30%) | 4 (22.22%) | | | | 16 (28.07%) | 4 (25.00%) | | |
